# Alcohol consumption screening of newly-registered patients in primary care: a cross-sectional analysis

**DOI:** 10.3399/bjgp13X673720

**Published:** 2013-09-30

**Authors:** Zarnie Khadjesari, Louise Marston, Irene Petersen, Irwin Nazareth, Kate Walters

**Affiliations:** Primary Care and Population Health, University College, London.; Primary Care and Population Health, University College, London.; Primary Care and Population Health, University College, London.; Primary Care and Population Health, University College, London.; Primary Care and Population Health, University College, London.

**Keywords:** alcohol drinking, cross-sectional studies, ONS Opinions (Omnibus) survey, primary health care, The Health Improvement Network (THIN)

## Abstract

**Background:**

Although screening and brief intervention is effective at reducing alcohol consumption in primary care and is recommended by guidelines, there are numerous barriers to its delivery. Screening newly-registered patients for alcohol-use disorders provides an opportunity for systematic collection of alcohol consumption data.

**Aim:**

To examine how alcohol screening data are recorded in primary care, the extent to which they are recorded, and whether reported levels of consumption differ from general population data.

**Design and setting:**

Cross-sectional analysis, with data collected from patients in the year after registration.

**Method:**

Data on alcohol consumption were collected from The Health Improvement Network (THIN) primary care database from patients aged ≥18 years, newly registered with a general practice in 2007 to 2009, and compared with the Office for National Statistics Opinions (ONS Omnibus) survey.

**Results:**

A total of 292 376 (76%) of the 382 609 newly-registered patients had entries for alcohol consumption (units a week, Read Codes for level of consumption, and/or screening test). Only 25 975 (9%) were recorded as completing a validated screening test, most commonly AUDIT/AUDIT-C (16 004, 5%) or FAST (9419, 3%). Alcohol-use disorders are underreported in primary care (for example, higher risk drinking 1% males, 0.5% females) in comparison with the Opinions survey (8% males, 7% females).

**Conclusion:**

Alcohol screening data are collected from most patients within 1 year of registration with a GP practice; however, use of a validated screening test is rarely documented and alcohol-use disorders are underreported. Further efforts are needed to encourage or incentivise the use of validated tests to improve the quality of data collected.

## INTRODUCTION

Primary care has long been established as an ideal setting for screening and brief intervention for reducing alcohol intake, being the first point of contact with health services.^1^ There is substantial evidence, spanning more than 20 years, to support the use of screening and brief intervention in this setting,^2^ which has led to its advocacy in National Institute for Health and Care Excellence (NICE) UK guidance.^3^ Recent findings from a large UK multicentre, multisetting (including primary care) trial of screening and brief intervention suggest that screening should be universal, rather than targeted at patients deemed as high risk, to identify the largest number of people with alcohol-use disorders;^4^ where alcohol-use disorders are defined by NICE as covering a ‘wide range of mental health problems as recognised within the international disease classification systems (ICD-10, DSM-IV). These include hazardous and harmful drinking and alcohol dependence’,^3^,^5^ in other words drinking above recommended limits. Screening new registrants for alcohol-use disorders as part of new patient health check questionnaires in general practice provides an opportunity for systematic screening (albeit short of universal screening of all patients), and is more acceptable to patients when collected in the context of other health behaviours.^6^

In 2003, GPs in England identified only 2.1% of alcohol-use disorders when compared with population survey data.^7^ Lack of financial incentive is often cited as one of the key barriers to delivering screening and brief intervention in primary care.^6^,^8^–^12^ There is currently no financial incentive through the Quality and Outcomes Framework (QOF) to encourage GPs to screen for alcohol consumption; this is one of the criticisms raised by the Alcohol Health Alliance UK^13^ of the government’s alcohol strategy. However, since April 2008, general practices in England have been offered a small financial incentive for screening newly-registered adult patients for alcohol-use disorders as part of Clinical Directed Enhanced Services (DES).^14^ The DES reimburses practices that use abbreviated versions of the World Health Organization’s Alcohol Use Disorders Identification Test (AUDIT),^15^ namely the FAST or AUDIT-C.

To the best of the authors’ knowledge this is the first study that aims to determine how alcohol screening is recorded in primary care, and the extent to which this is happening in newly-registered patients in their first year with the practice in UK primary care. There were three specific objectives:

- Describe how alcohol is recorded in UK primary care data; that is, use of Read Codes, units of alcohol, and screening tests.
- Describe the recording of alcohol consumption in primary care by sociodemographic factors (age, sex, and social deprivation) and by region (strategic health authority for England and country for Wales, Scotland, and Northern Ireland).
- Compare the level of alcohol intake recorded in primary care with population data (the Opinions survey).

**How this fits in**

New patient health check questionnaires in general practice provide an opportunity for systematic screening for alcohol-use disorders. This study determines how alcohol screening is recorded in primary care, and the extent to which this occurs in newly-registered patients. Although alcohol consumption is recorded in 76% of newly-registered patients, it is rarely through documented use of validated screening tests. Alcohol-use disorders continue to be underreported in primary care compared with general population data.

## METHOD

### Data source

The Health Improvement Network (THIN)^16^ is a primary care database containing electronic patient records from over 500 general practices, covering approximately 6% of the UK population. The database contains details of symptoms, diagnoses, prescriptions, test results, and health indicators. Information can be entered into the database as free text or Read Codes. Read Codes are standardised across all UK general practices. THIN is broadly representative of the UK population in terms of age, sex, deprivation, and geographical distribution.^17^ THIN includes the Townsend deprivation index,^18^ which is a composite measure of social deprivation presented as quintiles.

To ensure that only data of an acceptable standard were included for analysis, data from individual practices were included in this study if their data were after the Acceptable Mortality Reporting (AMR)^19^ and Acceptable Computer Usage (ACU) dates.^20^ AMR and ACU are measures of the extent to which mortality data is entered on the computer and the computer is used for general recording of information respectively. Both markers are applied to the data from each general practice.

### Patient eligibility

This study included patients aged ≥18 years who registered with a general practice in 2007, 2008, and 2009. Alcohol screening data were used in this study if recorded in the year after registration. Patients leaving a practice within the first year of registration were excluded from the analysis.

### Read Codes and continuous measures of drinking

Three types of data were extracted from THIN and included in this study:

1. Read Codes for level of alcohol consumption;
2. Read Codes for types of screening test used; and
3. continuous measure of drinking (that is, units a week).

Read Codes that reflect levels of drinking were selected. These Read Codes typically represent a drinking category, for example moderate drinker 3–6 units a day, but also include codes that indicate drinking above limits; for example, hazardous or harmful drinking. Read Codes were also included that indicate the types of screening tool used to determine how many people had completed a validated screening test. Data on alcohol consumption as a continuous measure of the number of units consumed in a week were extracted. Where patients had more than one data entry, the earliest was selected for the data presented in Tables 1 and 2. These alcohol consumption screening data and information on patient age, sex, social deprivation, and geographical region were analysed using Stata (version 12).

### ONS Opinions (Omnibus) survey

Data on the prevalence of alcohol consumption were obtained from the Office for National Statistics (ONS) Opinions survey, Drinking Module.^21^ This survey collected data via face-to-face interviews with a sample of approximately 1200 adults aged ≥16 years (drinking data only available for people aged ≥18 years) in private households across Great Britain. Data were extracted from the alcohol modules April 2008, April 2009, and May 2009 (total sample size 3218), which covered the study period of 2007 to 2009. The number of units for each person was calculated using the method recommended by ONS.^22^ A response rate of 58% was achieved in both April and May 2009 Opinions (Omnibus) surveys. Responses were weighted so that their distribution across regions, age, and sex matched that of the population.

### Analysis

**Figure 1:**
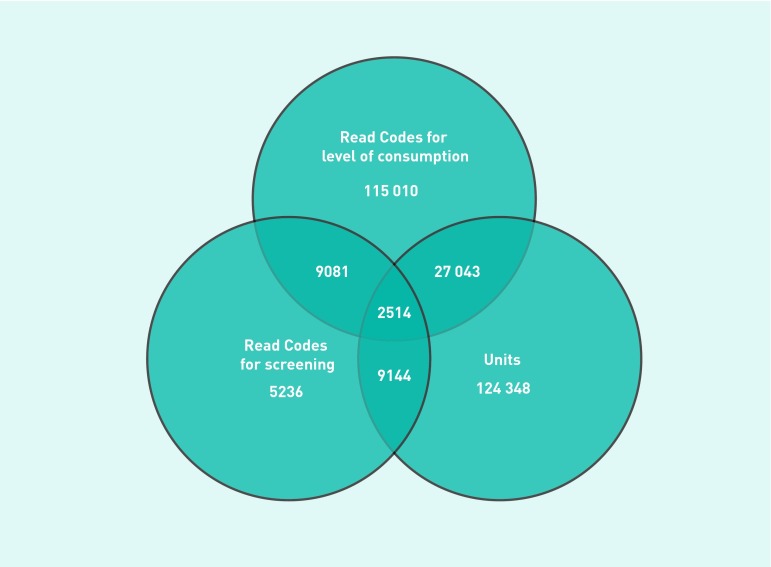
Venn diagram of the number of patients with Read Codes for level of consumption, Read Codes for screening tests, and continuous measures of drinking (units a week)

In the analysis of THIN data, descriptive data were used to illustrate the number (proportion) of patients with Read Codes for level of consumption, Read Codes for types of screening test used, and continuous measures of drinking. These data are presented graphically in Figure 1. Recording of alcohol consumption data (quantity measures and Read Codes for quantity) was described by region and sociodemographic factors. The relative ‘risks’ of a recording were examined by age, sex, region, and social deprivation using a Poisson regression analysis. In this analysis, region and social deprivation were treated as categorical variables. The effect of age was non-linear, in other words, alcohol consumption did not increase or decrease with age and therefore age was included in the model as cubic splines and added an interaction term between age (< and ≥60 years) and sex.

Recording of alcohol consumption in THIN was compared with population data (the Opinions survey). This was undertaken separately for males and females in THIN and Opinions. To compare the data between THIN and Opinions, four alcohol consumption categories were created:

1. no alcohol (including teetotallers and ex-drinkers);
2. lower risk;
3. increasing risk; and
4. higher risk.

Teetotallers and ex-drinkers were combined as these categories could not be reliably separated in THIN or the Opinions survey.

## RESULTS

There were 382 609 newly-registered patients who had been registered for at least a year. A total of 292 376 (76%) patients had been screened for alcohol consumption, with data recorded as units of alcohol in a week (only) (124 348, 43%), a Read Code for units in a week (only) (115 010, 39%), a Read Code for type of screening test used (only) (5236, 2%), or more than one of these methods of recording (47 782, 16%) (Figure 1). Most of the 25 975 (9%) patients recorded as completing a validated screening test received the AUDIT (including the AUDIT-C, 16 004, 5%) or the FAST (9419, 3%), with a small number completing other screening tests (552, 1%).

Overall rates of recording were highest among females and individuals aged 60–69 years (Table 1). The rates of recording in females aged 18–59 years were higher than those in males, with the patterns reversing between 60 and ≥90 years (*P*<0.001 for interaction between age groups [≥60 versus <60]) (Table 1). Rates of recording increased with level of deprivation. The highest rates of recording were found in the North East, followed by London. The lowest rates were found in Northern Ireland (Table 1).

**Table 1:** Rates of recording of alcohol consumption by sex

**Variable**	**Males**		**Females**	
	**Rate/100**	**95% CI**	**Rate/100**	**95% CI**
**Age group, years**				

18–19	53.9	47.4 to 61.3	61.8	56.1 to 68.2
20–29	63.6	60.4 to 67.0	69.6	66.9 to 72.4
30–39	68.7	66.2 to 71.2	73.3	71.0 to 75.8
40–49	72.1	69.8 to 74.4	74.3	72.3 to 76.7
50–59	75.9	73.7 to 78.1	76.9	74.9 to 79.0
60–69	79.1	76.9 to 81.2	79.0	77.0 to 81.2
70–79	76.2	74.0 to 78.5	75.3	73.2 to 77.4
80–89	64.5	61.6 to 67.6	57.2	54.2 to 60.2
≥90	45.6	40.7 to 51.2	42.2	38.2 to 46.9

**Townsend quintile**				

1 (least deprived)	66.9	63.8 to 70.1	69.9	67.0 to 72.9
2	67.8	65.2 to 70.4	70.7	68.2 to 73.3
3	68.8	66.2 to 71.4	71.3	68.8 to 73.8
4	68.9	65.9 to 72.1	71.4	68.2 to 74.6
5 (most deprived)	69.7	64.7 to 75.1	72.7	68.6 to 77.2

**Strategic health authority/country**				

Northern Ireland	54.0	40.7 to 72.6	61.8	48.4 to 79.3
South West	63.0	56.9 to 70.0	64.9	59.2 to 71.3
South East Coast	63.0	53.6 to 74.3	67.7	59.3 to 77.5
Scotland	64.7	59.0 to 71.2	66.8	61.0 to 73.4
South Central	65.3	59.5 to 71.8	68.4	62.5 to 74.8
Yorkshire and Humber	67.7	57.4 to 80.3	71.1	60.5 to 83.9
East Midlands	68.8	58.9 to 80.8	72.6	64.4 to 82.3
West Midlands	70.0	57.0 to 85.5	73.5	63.1 to 85.6
North West	71.0	64.7 to 78.1	73.4	66.9 to 80.7
East of England	71.2	66.0 to 78.3	73.2	66.5 to 80.7
Wales	71.3	63.6 to 80.5	73.5	65.4 to 83.2
London	76.1	70.4 to 82.3	78.3	72.4 to 84.7
North East	81.1	75.4 to 87.8	84.5	79.7 to 90.3

Of those patients with an alcohol consumption record in primary care, 21% of males and 31% of females reported no alcohol consumption or had Read Codes for teetotal/ex-drinker. This was nearly double the rate of those who reported no alcohol consumption in the Opinions survey (Table 2). In contrast, a much smaller proportion of those with a record of alcohol consumption in primary care reported being of increasing or higher risk. Notably only 2% of males and 1% of females were in the higher risk category in THIN, whereas 8% of males and 7% of females in the Opinions survey were reported as higher risk (Table 2).

**Table 2:** Numbers and percentages recorded in given alcohol consumption categories for THIN and the Opinions Survey by sex.

**Alcohol consumption categories**	**THIN**						**Opinions^d^**			
	**Males**			**Females**			**Males**		**Females**	
	***n***	**Total, % (95% CI)**	**Recorded, % (95% CI)**	***n***	**Total, % (95% CI)**	**Recorded, % (95% CI)**	***n***	**% (95% CI)**	***n***	**% (95% CI)**
***N***		181 653	133 919		200 956	153 221	1440		1778	
**Teetotal/ ex-drinker**	28 414	16 (15 to 16)	21 (21 to 21)	47 320	24 (23 to 24)	31 (31 to 31)	169	11 (10 to 13)	314	17 (16 to 29)
**Lower risk^a^**	92 287	51 (51 to 51)	69 (69 to 69)	97 690	49 (48 to 49)	64 (64 to 64)	896	63 (61 to 66)	1048	58 (56 to 61)
**Increasing risk^b^**	10 964	6 (6 to 6)	8 (8 to 8)	7225	4 (4 to 4)	5 (5 to 5)	269	18 (16 to 20)	301	18 (16 to 20)
**Higher risk^c^**	2254	1 (1 to 1)	2 (2 to 2)	986	0.5 (0.5 to 0.5)	1 (1 to 1)	106	8 (6 to 10)	115	7 (5 to 8)
**Not recorded**	47 734	26 (26 to 26)		47 735	24 (24 to 24)		0		0	

a Lower risk drinkers: drinking within recommended weekly limits of 1–14 units/week (females) and 1–21 units/week (males).

b Increasing risk drinkers: drinking above recommended weekly limits, but not above higher risk levels 15–35 units/week (females) and 22–50 units/week (males).

c Higher risk drinkers: drinking above this level causes the highest risk of harm ≥36 units/week (females) ≥51 units/week (males).

d Numbers are actual numbers responding to the survey, percentages and 95% CIs relate to weighted data.

## DISCUSSION

### Summary

Alcohol screening data were recorded for 76% of adult patients in the first year of registration with their GP practice from 2007 to 2009. These data were recorded as units of alcohol in a week (43%), Read Codes for levels of consumption (39%), Read Codes for type of screening test used (2%), or a combination of more than one of these approaches (16%). Recording of alcohol consumption data was most prevalent in female patients, 60–69 year olds, those who are more deprived, and patients in the North East of England and London. The primary care data had a higher proportion of patients reporting no consumption of alcohol and a lower proportion reported as increasing or higher risk drinkers, compared with population survey data.

Although it is encouraging that 76% of newly-registered adult patients in THIN have been asked about their alcohol consumption, it is unclear how these data were collected when use of a validated screening test was not recorded. Patients may be asked to report how much alcohol they consumed over the past week (actual consumption), or to estimate how much they consumed in a typical week (typical consumption). Reporting actual consumption is an easier task and leads to more accurate recall; however, this approach may overestimate the proportion of abstainers among occasional drinkers.^23^,^24^ The mode of assessment may also have an impact on the accuracy of the data collected, where patients are less likely to report sensitive behaviours face-to-face, compared with self-completed paper-based questionnaires, thus introducing social desirability bias.^25^ Alcohol consumption data can be recorded by self-completed questionnaires at registration or during face-to-face assessments, and it is not known which of these are used by the different general practices. The difference between modalities may, however, be less pronounced in the general practice setting where responses to either mode of assessment have potential repercussions; for example, are recorded in patient notes. Finally, it is unknown whether patients are given guidance on estimating a standard drink or unit of alcohol, depending on the size and strength of different drinks. This is important, as in 2009, around one-third of regular beer drinkers and one-sixth of regular wine drinkers were unsure of the number of units contained within these drinks.^26^

Higher rates of alcohol consumption recording were found in female patients aged 18–59 years who registered with a new practice, compared with male patients aged 18–59 years. Collection of alcohol screening data in the form of a paper-based questionnaire on registration with a practice should prevent sex disparities in screening. However, where data are collected by GPs there could be a variety of explanations for these differences. Females are more likely to consult than males,^27^ and therefore there could be more opportunistic recording of alcohol consumption. This study found fairly substantial regional variations in rates of screening for alcohol consumption in primary care. This regional variation is not explained by differences in the prevalence of hazardous drinking across the country, where highest rates of recording in this study were found in the North East, yet the highest prevalences of alcohol-use disorders in the population are found in the North West, Yorkshire and Humber, and the East Midlands.^28^

Only 9% of patients with alcohol screening data were recorded as completing a validated screening questionnaire (that is, had a Read Code for a screening test) despite the financial incentive offered to practices that record screening patients with a validated test. Nevertheless, of those patients who did complete a validated screening test, most (98%) completed either the AUDIT (including the AUDIT-C) or the FAST, as advocated since April 2008.^14^ It has been argued that the financial incentive of £2.33 for each screened patient (the value of DES incentive during the period of data collection) is too low to have an impact on clinical practice. Some Locally enhanced services (LES) offer increased financial incentives, which could explain some of the regional variation in screening. Researchers and clinicians are urgently calling for screening and brief intervention for alcohol-use disorders to be included in the Quality and Outcomes Framework as this is more likely to have an impact on clinical behaviour.^10^,^12^

### Strengths and limitations

This study provides an insight into how alcohol screening data are being collected in primary care using THIN, which covers approximately 6% of the UK population and is broadly representative of the UK population.^29^

It is important to note that newly-registered patients, who constitute around 15% of a typical practice list size, tend to be younger than the total practice population. This has implications for interpretation of the comparison of rates of reporting in primary care with those in the population-based Opinions survey. However, alcohol consumption is much more likely to be recorded in newly-registered patients than at any other time (only about 20% have further alcohol consumption recorded in subsequent years in primary care [I Petersen, personal communication, 2013). Further, subsequent recording of alcohol consumption is likely to be associated with the health status of individual patients.

The results of validated alcohol screening tests are entered on the Vision software (the software used by GP practices providing data to THIN) using free text, preventing interpretation of the score and use of the information on level of risk in this study. As only 2% of patients had screening test data alone, inclusion of these data probably would not have altered the findings substantially. However, as practices are encouraged to screen patients with validated screening tests, these data will become ever more valuable to researchers.

### Comparison with existing literature

Higher rates of no alcohol consumption and lower rates of increasing and higher risk drinking were reported in THIN, compared with the Opinions survey. This finding is congruent with the Cheeta study, published 10 years ago, where GPs in England identified only 2.1% of alcohol-use disorders when compared with population survey data.^7^ Also, the discrepancy could be more pronounced as population surveys are thought to account for only 60% of alcohol sales data in England, where missing data are attributed to underreporting and people not captured by the surveys, such as the institutionalised and non-responders.^30^

### Implications for practice

These findings show that practices are systematically recording data on alcohol consumption for most newly-registered patients; however, there is little evidence of the widespread use of validated screening tests to collect this information. Moreover, comparisons with a population survey suggest differences in the levels of alcohol consumption recorded in GP data, with an underrecording of alcohol-use disorders and an overrecording of no drinking in GP records in comparison with survey data. Practices should be encouraged to screen newly-registered patients for alcohol consumption using validated screening tests, such as the AUDIT-C or FAST, as recommended by NICE. This could be implemented with relatively little extra work for practices, as alcohol consumption data are already being collected for most newly-registering patients.

## Open access

This is an Open Access article distributed in accordance with the CC BY-NC 3.0 license http://creativecommons.org/licenses/.

## References

[1] Deehan A, Marshall EJ, Strang J (1998). Tackling alcohol misuse: opportunities and obstacles in primary care. Br J Gen Pract.

[2] Kaner EF, Beyer F, Dickinson HO (2007). Effectiveness of brief alcohol interventions in primary care populations. Cochrane Database Syst Rev.

[3] National Institute for Health and Clinical Excellence (2010). Alcohol-use disorders: preventing the development of hazardous and harmful drinking (PH24).

[4] Coulton S, Drummond C, Deluca P (2012). The utility of different screening methods to detect hazardous drinking and alcohol use disorders in the Screening and Intervention Programme for Sensible drinking (SIPS) programme. Addiction Sci Clin Pract.

[5] Babor TF, Higgins-Biddle JC (2001). Brief Intervention for hazardous and harmful drinking: a manual for use in primary care.

[6] Hutchings D, Cassidy P, Dallolio E (2006). Implementing screening and brief alcohol interventions in primary care: views from both sides of the consultation. Primary Health Care Res Develop.

[7] Cheeta S, Drummond C, Oyefeso A (2008). Low identification of alcohol use disorders in general practice in England. Addiction.

[8] Johnson M, Jackson R, Guillaume L, Meier P, Goyder E (2011). Barriers and facilitators to implementing screening and brief intervention for alcohol misuse: a systematic review of qualitative evidence. J Public Health.

[9] McAvoy BR, Donovan RJ, Jalleh G (2001). General practitioners, prevention and alcohol — a powerful cocktail? Facilitators and inhibitors of practising preventive medicine in general and early intervention for alcohol in particular: a 12-nation key informant and general practitioner study. Drugs Education Prevention Policy.

[10] Rapley T, May C, Frances Kaner E (2006). Still a difficult business? Negotiating alcohol-related problems in general practice consultations. Soc Sci Med.

[11] Wutzke SE, Donovan RJ, Gomel MK (1998). Enhancing the delivery of brief interventions for hazardous alcohol use in the general practice setting: a role for both general practitioners and medical receptionists. Health Promotion J Australia.

[12] Wilson GB, Lock CA, Heather N (2011). Intervention against excessive alcohol consumption in primary health care: a survey of GPs’ attitudes and practices in England 10 years on. Alcohol Alcoholism.

[13] Alcohol Health Alliance UK (2012). Inquiry into The Government’s Alcohol Strategy. http://www.rcplondon.ac.uk/sites/default/files/aha-submission-to-health-committee-inquiry-into-alcohol-strategy-2012.pdf.

[14] British Medical Association (2009). Clinical directed enhanced services (DESs) for GMS contract 2008/09 Guidance and audit requirements.

[15] Babor TF, Higgins-Biddle JC, Saunders JB, Monteiro MG (2001). AUDIT — The Alcohol Use Disorders Identification Test: guidelines for use in primary care.

[16] The Health Improvement Network http://csdmruk.cegedim.com/our-data/our-data.shtml.

[17] Blak BT, Thompson M, Dattani H, Bourke A (2011). Generalisability of The Health Improvement Network (THIN) database: demographics, chronic disease prevalence and mortality rates. Informatics Primary Care.

[18] Townsend P, Phillimore P, Beattie A (1988). Health and deprivation: inequality and the North.

[19] Maguire A, Blak BT, Thompson M (2009). The importance of defining periods of complete mortality reporting for research using automated data from primary care. Pharmacoepidemiol Drug Saf.

[20] Horsfall L, Walters K, Petersen I (2013). Identifying periods of acceptable computer usage in primary care research databases. Pharmacoepidemiol Drug Saf.

[21] Office for National Statistics (2010). ONS Opinions Survey, Drinking Module, April 2008 and April and May 2009.

[22] Goddard E (2007). Estimating alcohol consumption from survey data: updated method of converting volumes to units.

[23] Rehm J (1998). Measuring quantity, frequency, and volume of drinking. Alcoholism Clin Exp Res.

[24] Dawson DA (2003). Methodological issues in measuring alcohol use. Alcohol Res Health.

[25] Tourangeau R, Yan T (2007). Sensitive questions in surveys. Psycholog Bull.

[26] Lader D, Steel M (2010). Opinions Survey Report No. 42. Drinking: adults’ behaviour and knowledge in 2009.

[27] Wang EA (2013). Gender differences in consultation pattern in general practice: an analysis of routinely collected UK general practice data. PloS One.

[28] The NHS Information Centre (2009). Statistics on alcohol: England, 2009.

[29] Bourke A, Dattani H, Robinson M (2004). Feasibility study and methodology to create a quality-evaluated database of primary care data. Informatics Primary Care.

[30] Boniface S, Shelton N (2013). How is alcohol consumption affected if we account for under-reporting? A hypothetical scenario. Eur J Public Health.

